# Cysteine Pathogenic Variants of PMM2 Are Sensitive to Environmental Stress with Loss of Structural Stability

**DOI:** 10.1155/2023/5964723

**Published:** 2023-01-25

**Authors:** Fan Yu, Li Lin, Jingmiao Sun, Jicheng Pan, Yixin Liao, Yunfan Pan, Guannan Bai, Liangjian Ma, Jianhua Mao, Lidan Hu

**Affiliations:** ^1^The Children's Hospital, Zhejiang University School of Medicine, National Clinical Research Center for Child Health, Hangzhou 310052, China; ^2^Hubei Normal University, Huangshi, 435002 Hubei, China; ^3^Department of Obstetrics and Gynecology, Nanfang Hospital, Southern Medical University, Guangzhou 510515, China; ^4^Beijing Advanced Innovation Center for Structural Biology, Tsinghua-Peking Center for Life Sciences, School of Life Sciences, Tsinghua University, Beijing 100084, China; ^5^The First Clinical Medical College of Lanzhou University, Lanzhou 730000, China; ^6^Department of Nephrology, The Children's Hospital, Zhejiang University School of Medicine, National Clinical Research Center for Child Health, National Children's Regional Medical Center, Hangzhou, China

## Abstract

Congenital disorders of glycosylation (CDG) are severe metabolic disorders caused by an imbalance in the glycosylation pathway. Phosphomannomutase2 (PMM2-CDG), the most prevalent CDG, is mainly due to the disorder of PMM2. Pathogenic variants in cysteine have been found in various diseases, and cysteine residues have a potential as therapeutic targets. PMM2 harbor six cysteines; the variants Cys9Tyr (C9Y) and Cys241Ser (C241S) of PMM2 have been identified to associate with CDG, but the underlying molecular mechanisms remain uncharacterized. Here, we purified PMM2 wild type (WT), C9Y, and C241S to investigate their structural characteristics and biophysical properties by spectroscopic experiments under physiological temperature and environmental stress. Notably, the variants led to drastic changes in the protein properties and were prone to aggregate at physiological temperature. Meanwhile, PMM2 was sensitive to oxidative stress, and the cysteine pathogenic variants led to obvious aggregate formation and a higher cellular apoptosis ratio under oxidative stress. Molecular dynamic simulations indicated that the pathogenic variants changed the core domain of homomeric PMM2 and subunit binding free energy. Moreover, we tested the potential drug targeting PMM2-celastrol in cell level and explained the result by molecular docking simulation. In this study, we delineated the pathological mechanism of the cysteine substitution in PMM2, which addressed the vital role of cysteine in PMM2 and provided novel insights into prevention and treatment strategies for PMM2-CDG.

## 1. Introduction

Congenital disorders of glycosylation (CDG) 2 are genetic disorders resulting from abnormal glycosylation. PMM2-CDG, due to the impairment of enzyme phosphomannomutase2 (PMM2), is the most prevalent CDG with an incidence rate of 1 in 20,000 individuals [1–3]. The clinical symptoms of PMM2-CDG involve multisystem disorders, such as hypotonia, stroke-like episodes, and peripheral neuropathy [4]. There is a substantial childhood mortality rate of approximately 15–30%, and surviving patients develop permanent neurological disabilities [5]. PMM2-CDG, considered a misfolding proteins disease, ongoing efforts are made to develop drugs, including glucose-1,6-bisphosphate, D-mannose, epalrestat, or proteostasis regulators [6–9]. Increasing efforts towards the treatment of misfolding proteins disease, such as identification some chaperones: small-molecule modulators or structural-correctors for conformationally destabilized proteins; PROTACs and autophagosome-tethering compound (ATTEC) to degrade misfolded proteins, DAXX to prevent aggregation, *etc* [10–13]. However, to date there is no specific curative treatment is available [14–16]. Therefore, elucidating the pathogenesis of PMM2-CDG is warranted and might provide new insights into its treatment.

PMM2 is highly conserved among species and essential for early development, and deficiency of PMM2 led to lethality in a mouse and zebrafish model [17, 18]. PMM2 catalyzes mannose-6-phosphate to the mannose-1-phosphate precursor of the GDP-mannose [1]. Very recently, the complete crystal structure of hPMM2 was resolved, including the cap domain (87-184 aa) and the core domain (1-83 and 189-246 aa), folding 9 *α*-helix, 11 *β*-sheet and two flexible linker regions (84-86 and 185-188 aa) [19]. Over 135-point pathogenic variants have been reported in *PMM2*, and approximately 85% of these pathogenic variants are missense (http://www.hgmdl.cf.ac.uk). When we examined the specific mutation sites of PMM2 in the clinical report, cysteine (Cys) pathogenic variants have attracted our attention. The primary structure of PMM2 comprises six Cys residues (C9, C83, C103, C136, C192, and C241), and four (C9Y, C103F, C192G, and C241S) of these sites are linked to PMM2-CDG [20–22].

The Cys residues, as one of the least abundant amino acids, have unique attributes to the structure and function of proteins, especially for the catalytic activity and protein folding [23–25]. As the Cys residues likely have higher reactivity properties than other amino acids, replacement of even one such ‘critical' residue may lead to drastic changes in the protein's properties [26, 27]. Sulfur-containing amino acid residues, such as the Cys residues in proteins, are particularly sensitive to oxidative damage [28, 29]. However, in this case, the opposite is observed. The Cys residues are mutated to other amino acids that cause the disease. Thus, it is significantly important to delineate the uncommon role of Cys in PMM2.

Previous studies focused on detecting the enzymatic differences between WT and disease-associated variants [14, 15, 30]. There was no study focused on the effects of Cys variants on the structure of the PMM2 protein and cellular function. In this study, we selected two Cys variants that may form disulfide bonds to identify the role of the Cys residue in the protein structure and function in cells.

Our results showed that the C9Y and C241S destabilized the secondary and tertiary structures of PMM2, increased the susceptivity to oxidase stress and temperature, and promoted PMM2 protein aggregation and degradation, which eventually induced cell death. Findings from this study will help elucidate the molecular mechanism underlying the loss-of-function of the Cys pathogenic variants in PMM2 and facilitate the development of personalized PMM2-CDG treatment strategies.

## 2. Materials and Methods

### 2.1. Materials

DNA polymerase, restriction endonucleases, and DNA ligase were purchased from Takata. Dimethyl sulfoxide (DMSO), paraformaldehyde (PFA), Triton X-100, Nonidet P40 (NP-40), phenylmethanesulfonyl fluoride (PMSF), heparin, protease inhibitor cocktail, KCl, MgCl_2_, DTT, EDTA, Imidazole, cisplatin, H_2_O_2_, isopropyl-1-thio-*β*-D-glucopyranoside (IPTG), and 1-anilinonaphthalene-8-sulfonate (ANS) were Sigma products. *Escherichia coli*. (*E. coli*) DH5*α* and BL21 (DE3) strains were obtained from Biomed (Beijing, China). Dulbecco's modified Eagle's medium (DMEM), fetal bovine serum (FBS), lipofectamine 2000, and DAPI were purchased from Invitrogen. The antibody against PMM2 and p62 were obtained from Abcam, while the antibodies against GFP and GAPDH were from Yeasen Biotechnology. Donkey anti-rabbit Alexa Fluor 549, HRP-conjugated affinipure goat anti-rabbit IgG (H+L) were from EarthOx. BSA and skim milk powder were from BD Biosciences. All other chemicals were local products of analytical grade.

### 2.2. Plasmid and Site-Directed Mutagenesis

The open reading frame of *PMM2 (NM_000303)* was purchased by Youbio Co., Ltd. For the prokaryotic system, the WT PCR primers were as follows: Forward (F): 5′- ATTCGAGCTCCGTCGACATATGGCAGCGCCTGGCCCAGCGCTCT-3′; Reverse (R): 5′- TGCTCGAGTGCGGCCGCTTAGGAGAACAGCAGTTCACAG-3′. The C9Y variant was constructed by PCR-based site-directed mutagenesis: F, 5′-TCAGATCTCGAGCTCAAGCTTATGGCAGC GCCTGGCCCAGCGCTCTAC-3′; R, the same with WT. The C241S variant was constructed by PCR-based site-directed mutagenesis: F, the same with the WT; R, 5′- TGCTCGAGTGCGGCCGCTTAGGAGAACAGCAGTTCACTGATCC-3′. Then, PCR product was inserted into the expression pET28a(+) plasmid containing 6x His-tag. The recombinant plasmids containing the WT or mutation gene was transformed into *E. coli* BL21 (DE3) for the overexpression of the recombinant protein. For the eukaryotic system, the WT PCR primers were as follows: Forward (F): 5′- TCAGATCTCGAGCTCAAGCTTATGGCAGCGCCTGGCCCAGCGCTCT-3′; Reverse (R): 5′- CGACTGCAGAATTCGAAGCTTGGAGAACAGCAGTTCACAGATCC-3′. The C9Y variant was constructed by PCR-based site-directed mutagenesis: F, 5′- TCAGATCTCGAGCTCAAGCTTATGGCAGCGCCTGGCCCAGCGCTCTA c-3′; R, the same with WT. The C241S variant was constructed by PCR-based site-directed mutagenesis: F, the same with WT; R, 5′-CGACTGCAGAATTCGAAGCTTGGAGAACAGCAGTTCACTGATCC-3′. The obtained gene was inserted into the pEGFP-C3 vector, and endotoxin-free plasmids for cell transfection were obtained using the Plasmid Maxiprep kit.

### 2.3. Expression and Purification of Recombinant PMM2

Details about expression and purification were conducted by the same methods as those described previously [14]. The *E. coli* BL21 (DE3) containing the WT or variants' plasmids were amplified in the Luria-Bertani medium. The *E. coli* cells were harvested, lysed, and the soluble fraction were obtained by centrifugation at 12000 g at 4°C for 0.5 hours. After filtration 0.22 *μ*m pore size filter twice, the recombinant protein was collected using a Ni-NTA affinity column and purified by Hiload 16/600 Superdex 75 preparative column equipped with ÄKTA purifier in buffer containing 20 mM Na_2_HPO_4_, 150 mM NaCl, and 1 mM EDTA. The protein concentration was determined according to the BCA method.

### 2.4. Size Exclusion Chromatography (SEC), SDS-PAGE Analysis, and Protein Solubility

The purity of the final protein (>98%) was checked through SDS-PAGE and size exclusion chromatography (SEC) analysis and stored at -80°C. The SEC analysis was performed using ÄKTA purifier in the buffer with an elution rate of 0.6 mL/min. About 100 *μ*L protein solutions were injected into the column. The protein concentration for SEC analysis was 0.2 mg/mL. The SDS-PAGE analysis was performed using the 10% separating gel. About 10 *μ*L protein solutions with a protein concentration of 0.2 mg/mL were used for the SDS-PAGE analysis. Size exclusion chromatography can be used to estimate the molecular weight (MW) [31–33]. The purified protein was concentrated by Millipore Amicon Ultra-15 series and Millipore Amicon Ultra-0.5 series concentrators at 11000xg on 4°C. The protein concentration was determined every 15 min until the maximum concentration was reached. The maximum protein concentration was defined by the unchanged value after three successive replications of centrifugation. All 7solubility experiments are performed at least in triplicate.

### 2.5. Spectroscopy Experiments

Details about spectroscopic experiments were conducted by the same methods as those described previously [34]. In brief, the fluorescence spectra was determined using the F-4700 fluorescence spectrophotometer (Hitachi Co., Tokyo, Japan). The intrinsic fluorescence was monitored with an excitation wavelength of 280 nm or 295 nm, respectively, and an emission wavelength from 300 to 400 nm. For extrinsic ANS fluorescence measurement, the excitation wavelength was 380 nm and the scanning wavelength ranged from 400 to 700 nm. The Far-UV circular dichroism (CD) experiments were measured using Jasco J-715 spectropolarimeter (Jasco Corp, Tokyo, Japan) at room temperature using 0.2 mg/mL protein concentrations, respectively. Far-UV CD signals were collected using a 1 mm path length cell over a wavelength range of 190–250 nm. The solution turbidity was detected by the absorbance at 400 nm with an Ultraspec 4300 pro UV-Vis spectrophotometer (Amersham Pharmacia Biotech). The parameter *A*, defined as the ratio of the intensity at 320 nm (*I*_320_) to 365 nm (*I*_365_), suggested the position and shape change of the Trp fluorescence spectrum [35]. All spectroscopy experiments were repeated at least three times, and the buffer control was subtracted for correction.

### 2.6. Free Thiol Measurement

The number of free thiol in the proteins was determined by a micrototal mercapto assay kit (Solarbio, product number: BC1375). The thiol groups react with 5,50-dithiobis-(2-nitrobenzoic acid) (DTNB) and form yellow compounds with a maximum absorption peak at 412 nm. Evolution 300 Security UV-Vis Spectrophotometer (Thermo Fisher Scientific, Madison, USA) was used to detect the absorption peak at 412 nm, and then, the number of free thiols was calculated according to the formula given in the assay kit.

### 2.7. Cell Culture, Cell Transfection, and Immunofluorescence

HEK293T cells were obtained from public cell banks (ATCC, USA). These cells were cultured in DMEM containing 4.5 g/L high glucose, 10% fetal bovine serum, and 1% penicillin/streptomycin (Solarbio Science & Technology). All the cells were incubated in a humidified 37°C incubator and 5% CO_2_. All the recombinant plasmids (WT, C9Y, and C241S) were transiently transfected in HEK293T cells by Hieff Trans™ Liposomal Transfection Reagent (Yeason), following the manufacturer's protocol. After 24 h transfection, for the oxidative stress group, the cells were treated with 2 mM H_2_O_2_ for 2 h. Then, the two groups of cells were washed by PBS buffer three times for 5 min, fixed by 4% PFA for 40 min, treated by 0.4% Triton X-100 for 10 min, and blocked by 10% FBS for 40 min. The fixed cells were stained by P62 antibody. The nuclei were dyed with DAPI. The cells were observed by OLYMPUS IX83-FV3000-OSR confocal microscope. The percentage of cells with aggregates was qualified by calculating the percentages from at least 200 positively transfected cells from 5 random fields.

### 2.8. Cell Apoptosis Assay

Untreated or treated cells were detached using Trypsin-no EDTA and collected by 300 g for 5 min and washed with cold PBS twice at 4°C. The procedure was followed by the instruction of Annexin V-Alexa Fluor 647/PI Apoptosis Detection Kit. Cell analysis was performed on BD FACS Calibur flow cytometer (BD Biosciences) within 1 h. All experiments were performed in triplicate.

### 2.9. Cell Viability

The Cell Counting Kit-8 (Solarbio Science & Technology) was performed to detect cell proliferation. The 3^∗^10^3^ cells in 96-well plates were treated with 2 mM H_2_O_2_ for 2 h. 10 *μ*L CCK8 reagent was added into each well according to the instruction of CCK8. And then, absorbance of samples was detected at 450 nm wavelength using an MD M5 (molecular devices).

### 2.10. Western Blot

The cells transfected for WB were divided into four groups based on different treatments: normal, 2 mM H_2_O_2_, 0.5 *μ*M celastrol, and DMSO. The total protein concentration was detected using BCA Protein Quantification Kit (Vazyme). The nonreducing SDS-PAGE was done following the same protocol of the normal SDS-PAGE analysis without *β*-mercaptoethanol.

### 2.11. Molecular Dynamic Simulations

This study used the X-ray structure of the human PMM2 dimer template with residues 7–245 at pH 7.0 (PDB ID: 7O0C). The structure of the PMM2-C9Y and C241S dimer were constructed based on WT by PyMOL. Details of molecular dynamic (MD) simulation analysis were the same as those described previously. In brief, all structures were immersed in cubic water box with 10 Å between protein and the box boundary and water box contained 150 mM NaCl and 5 mM MgCl_2_. Calculations were simulated by GROMACS in CHARMM36 force field. Water was described with the TIP3P model. Electrostatics were treated using the particle mesh Ewald (PME) method We equilibrated the system for 5 ns under NVT and NPT conditions at 300 K and ran program for 100 ns to generate trajectories. Finally, visual molecular dynamics (VMD) was used to process and analyze the trajectories.

### 2.12. Protein Disulfide Bond Determination by Mass Spectrometry (MS)

Related principles and procedures were referred from Gorman et al. [36]. This study used 50 *μ*L PMM2-WT protein solution with a concentration of 0.2 *μ*g/*μ*L. We added prewashed beads and ethanol and elution the protein sample. Digestion the sample with trypsin+lysC mix (trypsin : protein = 1 : 50 (*w*/*w*)), and samples were freeze-dried in vacuum concentrator and redissolved with 0.1% trifluoroacetic acid. The sample was further analyzed by matrix-assisted laser desorption/ionization (MALDI) and electrospray ionization (ESI) in MS platform of Westlake University Institute for advanced study.

### 2.13. Data Analysis and Visualization

Data were analyzed by GraphPad Prism 8.0 (GraphPad Software Inc., San Diego, CA, USA). A *p* value of less than 0.05 was considered statistically significant. Results were visualized by GraphPad prism 8.0. All experiments in this work were repeated three times.

## 3. Results

### 3.1. Cys Substitution Impaired the Secondary and Tertiary Structures of PMM2 and Decreased Protein Solubility

**Figure 1:**
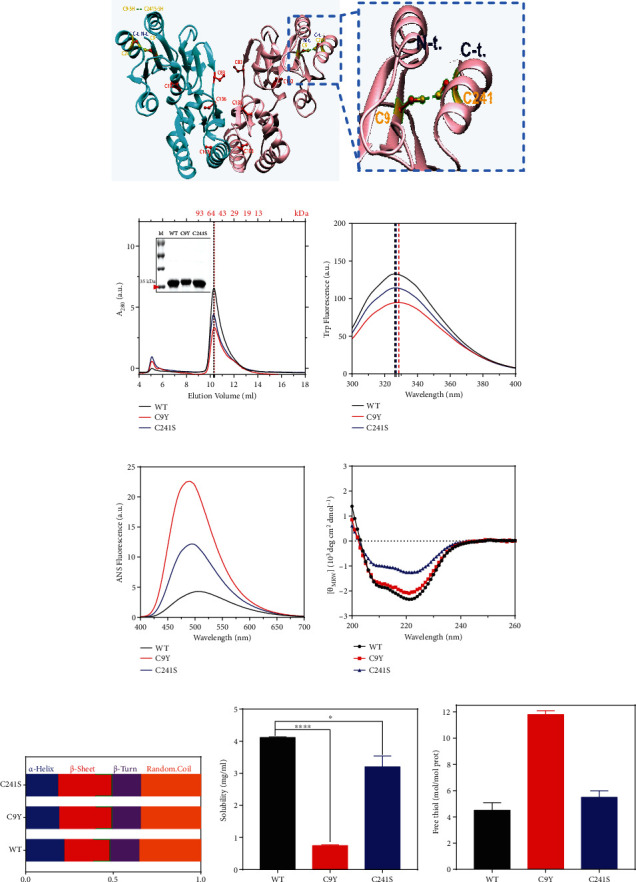
The cysteine variants decreased protein solubility with changing the secondary and tertiary structures of PMM2. (a) Representative cartoon crystal structure of the hPMM2 dimer (PDB ID: 7O0C). All the Cys were marked by stick, C9 and C241 colored by orange and the others colored by red. (b) SEC profiles of 100 *μ*L, 0.2 mg/mL and SDS-PAGE analysis of the purified proteins. The inset shows the SDS-PAGE analysis of the purified proteins. (c) Intrinsic Trp fluorescence spectra of the 10 *μ*M WT, C9Y, and C241S. (d) ANS fluorescence spectra of the 10 *μ*M WT, C9Y, and C241S. (e) Far-UV CD spectra of the 10 *μ*M WT, C9Y, and C241S. (f) Qualification the secondary ratio according to the Far-UV CD spectra by CDNN software. (g) The solubility of WT, C9Y, and C241S at 4°C (^∗^*p* < 0.05). (h) Number of free thiol of WT C9Y and C241S, the DTNB-reactive free −SH groups were calculated using a standard curve determined using L-cysteine. The presented results were calculated from three repetitions (^∗∗∗∗^*p* < 0.0001).

The C9Y (c. G26A) variant has been identified in at least 7 families from France, Germany, Sweden, and the USA [37, 38]. The C9 was highly conserved and located near the active site Asp12, probably leading to enzymatic inactivation. And the C241S (c. G722C) variant has been identified in at least 6 families from France, Belgium, Spain, and the USA [3, 21, 22]. In terms of the enzyme, the C9Y variant retained approximately 28% of the enzymatic activity of the WT, and the C241S variant retained approximately 32% [30]. The C9Y and C241S substitution were predicted to be “probably damaging” with scores of 0.969 and 0.987, respectively, by Polyphen analysis, which was consistent with the result predicted by PROVEAN (C9Y: -5.493, C241S: -2.986). Combining the structure from the PDB website (PDB ID: 7O0C) and bioinformation, we found that the distance between Cys9 and Cys241 of PMM2 was suitable for disulfide bond formation and MS have confirmed (Figure 1(a) and Table 1).

To elucidate the Cys substitution effect on the PMM2 protein, the recombinant WT, C9Y, and C241S proteins were purified from the *E. coli* expression system and measured by spectroscopic experiments. According to the results of SEC analysis and SDS-PAGE, a single main peak and band indicated that the purified proteins had high homogeneity (Figure 1(b)). The results showed that the C9Y variant had slight shift at elution volume, which suggested that it had a bigger apparent molecular weight, consistent with the SDS-PAGE result. The C241S variant had similar elution positions in the SEC profiles as WT, thereby suggesting that C241S mutation did not affect the overall molecular size, oligomeric state, or hydrodynamic radius of PMM2. According to analysis on the Superdex 75 10/300GL gel column, both the WT and variants had a molecular mass of ∼60 kDa in aqueous solution, which is approximately the theoretical homomer molar mass of 55.7 kDa. The catalytically active form of the PMM2 enzyme is a homodimeric protein, and we performed the assay and confirmed that the purified proteins had the enzyme activity (supplement Figure 1), meaning the purified proteins as dimmer form, consistent with a previous study [14, 19]. The effect of the Cys substitution on the microenvironment around Trp was further evaluated by the intrinsic Trp fluorescence. Compared to the WT protein, the Cys pathogenic variants dramatically decreased the Trp fluorescence of PMM2, and the C9Y was accompanied with an about 2 nm redshift of the maximum emission wavelength (*E*_max_), suggesting that the structure of Cys variants became loose (Figure 1(c)). The ANS spectra provided clear evidence that the Cys substitution induced a large change in the nonpolarity of the ANS-binding site, and the C9Y variant caused a larger change than the C241S variant did (Figure 1(d)). Far-CD spectra represent the protein secondary structure elements. In the Far-UV regions, the variants CD spectra were generally characterized by distinct peaks at 208 and 222 nm, respectively, which are features of proteins that contain *α*-helix conformational elements, displayed lighter 208 nm and 222 nm minima than the WT (Figure 1(e)). The ratio of *α*-helix and *β*-sheet of C9Y were significantly lower than that in WT after qualification by CDNN software (Figure 1(f)). Moreover, the solubility of the C9Y variant was decreased (WT: 4.12 ± 0.15 mg mL^−1^, C9Y: 0.75 ± 0.14 mg mL^−1^, and C241S: 3.21 ± 0.11 mg mL^−1^) (Figure 1(g)). Taking into account that the Cys substitution may affect the amount of free thiol, C9Y and C241S were monitored by DTNB modification according to Ellman's method. As expected, the C9Y variant contained more number of free thiol than the WT, which provided possible support for the disulfide-bonding network between the C9 and C241 (Figure 1(h)). Meanwhile, we performed mass spectrometry (MS) to detect the formation of disulfide bond. The lower value of score, the higher potential to form disulfide bond. The value below 1*E* − 3 was considered to form disulfide bond, and the value of C9 and C241 was 5.01*E* − 04 (Table 1). Taken together, these results support the hypothesis that Cys variants caused the changes in the secondary and tertiary structures of PMM2 that led to the Cys variants becoming more unstable.

### 3.2. Cys Substitution Impaired the Structural Stability at Physiological Temperature

**Figure 2:**
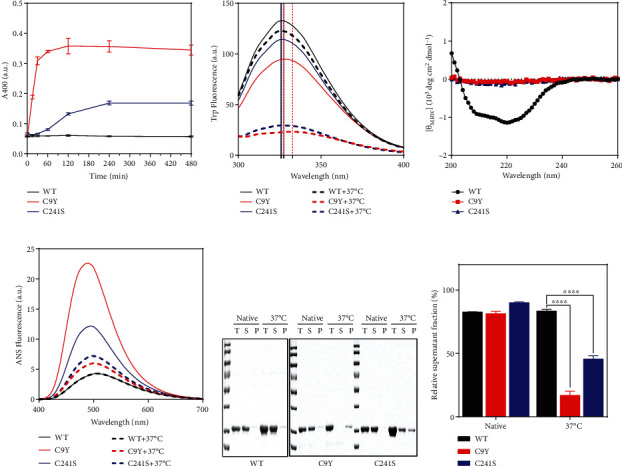
The C9Y and C241S variants lowered the stability of PMM2 under physiological temperature. (a) Turbidity (*A*_400_) curve of WT, C9Y, and C241S during under physiological temperature for 15 min, 30 min, 1 h, 2 h, 4 h, and 8 h. (b) Intrinsic Trp fluorescence spectra of the 10 *μ*M WT, C9Y, and C241S under physiological temperature for 4 h. (c) Far-UV CD of the 10 *μ*M WT, C9Y, and C241S under physiological temperature for 4 h. (d) ANS fluorescence spectra of the 10 *μ*M WT, C9Y, and C241S under physiological temperature for 4 h. (e) SDS-PAGE analysis of the soluble and insoluble fractions of protein solutions incubated at 37°C for 4 h. T: total, S: supernatant, P: precipitate. (f) Qualification of the soluble expression of proteins by SDS-PAGE analysis (e) with the supernatant/total (^∗∗∗∗^*p* < 0.0001).

To assess the effect of Cys substitution on protein stability, the aggregation of the samples was monitored by measuring the turbidity at 400 nm (*A*_400_) at a physiological temperature of 37°C at consecutive time points, where the optical density of the protein solution was used as a measurement of the protein aggregation. As the data showed that the *A*_400_ value of C9Y and C241S reached a plateau at 4 hour (h) and significantly higher than the WT (Figure 2(a)). Thus, the intrinsic Trp, extrinsic ANS fluorescence, and Far-CD spectra were monitored at the 4 h timepoint. It is intriguing to discover the Cys variants led to drastic structural change loss, characterized by a redshifted peak position and lower intensity (Figure 2(b)). Strikingly, major alterations of protein secondary structures were observed by Far-CD spectra (Figure 2(c)). In addition, the extrinsic ANS fluorescence was higher intensity and a blue-shifted peak position compared to the WT (Figure 2(d)). For a more intuitive and more comprehensive analysis, sedimentation assay was applied, in which samples were centrifuged, and partitioning of PMM2 into the soluble fraction was used as a measurement of the state of PMM2. According to the SDS-PAGE analysis, the C9Y variant remained mainly in the soluble fraction, whereas a vast amount of protein entered the pellet fraction after 4 h under 37°C treatment; for the C241S variant, almost half the protein entered the pellet fraction at 37°C treatment. However, WT remained in the soluble fraction even when all other conditions were the same (Figure 2(e); see Figure 2(f) for quantification). Together, these results demonstrate that the Cys substitution became more unstable and prone to aggregation.

### 3.3. The Mutation Increased PMM2 Susceptibility to Heat Shock

**Figure 3:**
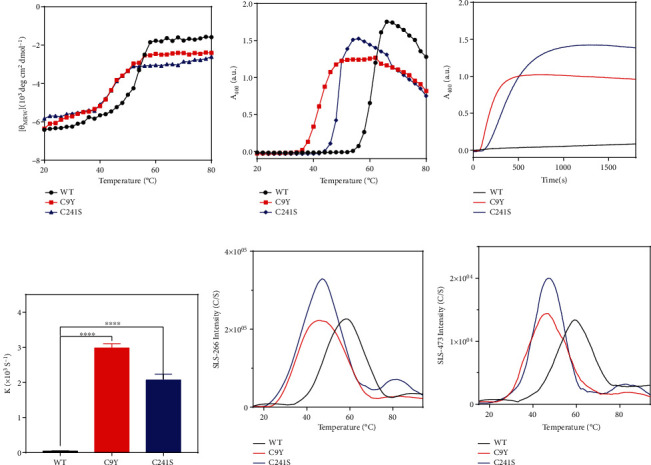
The C9Y and C241S variants significantly impairs thermal stability of PMM2. (a) The ellipticity at 222 nm of Far-UV CD upon temperature-gradient changes. (b) Turbidity (*A*_400_) curves of WT, C9Y and C241S during temperature-gradient heating experiments. (c) Thermal aggregation kinetics of the proteins obtained by heating the protein solutions at 50°C. (d) The lag time and aggregation rate (*k*) of thermal aggregation kinetics. (e, f) The Uncle system evaluated the temperature-gradient heating process with different protein concentrations. The emission intensity in 266 (e) and 473 nm (f) wavelength of WT, C9Y, and C241S with 10 *μ*M during the heating process, E266 and E473 with index of small and large aggregates, respectively (^∗∗∗∗^*p* < 0.0001).

We aimed to elucidate the thermal stability of the PMM2, for which we applied previously described procedures [34]. CD spectroscopy, *A*_400_, and Uncle were applied to obtain further information on the temperature sensitivity of the PMM2 WT and Cys variants. As shown in Figure 3, as expected, the Cys variants showed significantly decreased starting and midpoint temperatures of thermal denaturation (*T*_*m*_) when measured by the transition curves that were obtained from the changes of *E*_222_ in Far-UV CD (Figure 3(a)) and *A*_400_ (Figure 3(b)). In addition, the unfolding profiles were obviously different, and the heat denaturation analysis revealed that the WT began to dramatically lose the CD signal at 54°C, whereas the Cys variants started to significantly lose the CD signal at a temperature as low as 46°C for C9Y and C241S.

The *A*_400_ data of PMM2 were fit very well to a two-state thermal transition model between the folded and unfolded states and the *T*_*m*_ values were calculated. The *T*_*m*_ values of the WT, C9Y, and C241S proteins were 60.81 ± 0.1°C, 42.02 ± 0.2°C, and 48.72 ± 0.1°C, respectively. The thermal aggregation kinetics were determined by recording the time-course changes in turbidity when heating the protein solutions at 50°C (Figure 3(c)). The *A*_400_ values of Cys variants abruptly increased over time, but that of WT nearly sustained at baseline, implying that the variants aggregated with a shorter lag time and a faster aggregation rate (Figure 3(d)). Simultaneously, the Uncle was applied to monitor the more detail status of thermal unfolding and aggregation of WT and the Cys variants. The results of *E*_266_ and *E*_473_ were showed in Figures 3(e) and 3(f). The Cys variants each had a higher peak and lower temperature for the formation of small and large aggregates. Notably, these multifaceted results implied that the Cys pathogenic variants appeared to disrupt a compact domain organization and prone to forming more massive aggregates.

### 3.4. The Cys Substitution of PMM2 Caused More Aggregates and Higher Cellular Apoptosis under Oxidative Stress

**Figure 4:**
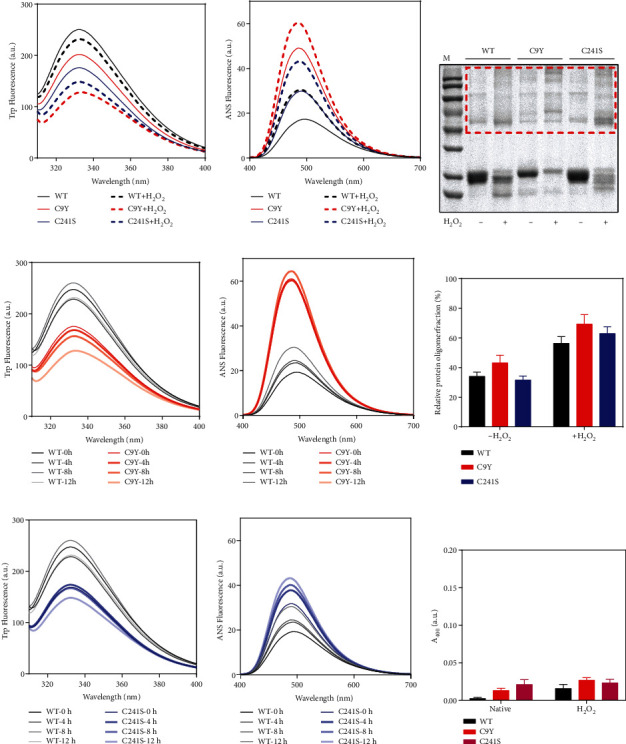
The C9Y and C241S variants elevated PMM2 susceptibility to H_2_O_2_ treatment. (a, b) Intrinsic Trp and ANS fluorescence spectra of the 10 *μ*M WT, C9Y, and C241S after 1 mM H_2_O_2_ treatment for 12 h at 4°C. (c, f) Nonreducing SDS-PAGE analysis of the protein solutions, the samples were incubated in SEC buffer with or without 1 mM H_2_O_2_ for 12 h. (d, e) Intrinsic Trp and ANS fluorescence spectra of the 10 *μ*M WT and C9Y after 1 mM H_2_O_2_ treatment for 4 h, 8 h, and 12 h. (g, h) Intrinsic Trp and ANS fluorescence spectra of the 10 *μ*M WT and C241S after 1 mM H_2_O_2_ treatment for 4 h, 8 h, and 12 h. (i) Turbidity (*A*_400_) curves of the 10 *μ*M WT, C9Y, and C241S after 1 mM H_2_O_2_ treatment for 12 h at 4°C.

The Cys residues and disulfide bonds were expected to exert reversal thiol oxidation effect upon temporary oxidative stress shock. In our cases, what effects did the Cys substitution and subsequent potential disulfide bond disruption on the protein structure upon temporary oxidative stress shock? We applied hydrogen peroxide (H_2_O_2_) induced oxidative stress. H_2_O_2_ played a clear role as signaling second messenger in the cell. The result in Figure 4 indicated that the purified Cys variant proteins had a redshifted peak position, lower intensity in the Trp fluorescence curve (Figure 4(a)), and higher intensity and a blueshifted peak position in extrinsic ANS fluorescence curve (Figure 4(b)). As shown in Figures 4(d)–4(g), the intensity of the Trp fluorescence reduced and the intensity of the ANS fluorescence enhanced along the time course of H_2_O_2_ treatment. Together, PMM2 proteins were sensitive to oxidative stress. However, the changed structures of Cys variant proteins did not cause aggregate formation showed by *A*_400_ curve (Figure 4(i)). Further, nonreducing SDS-PAGE results indicated that the changed structure of Cys variant proteins may exist as higher multimeric form (Figure 4(c); see Figure 4(f) for quantification).

**Figure 5:**
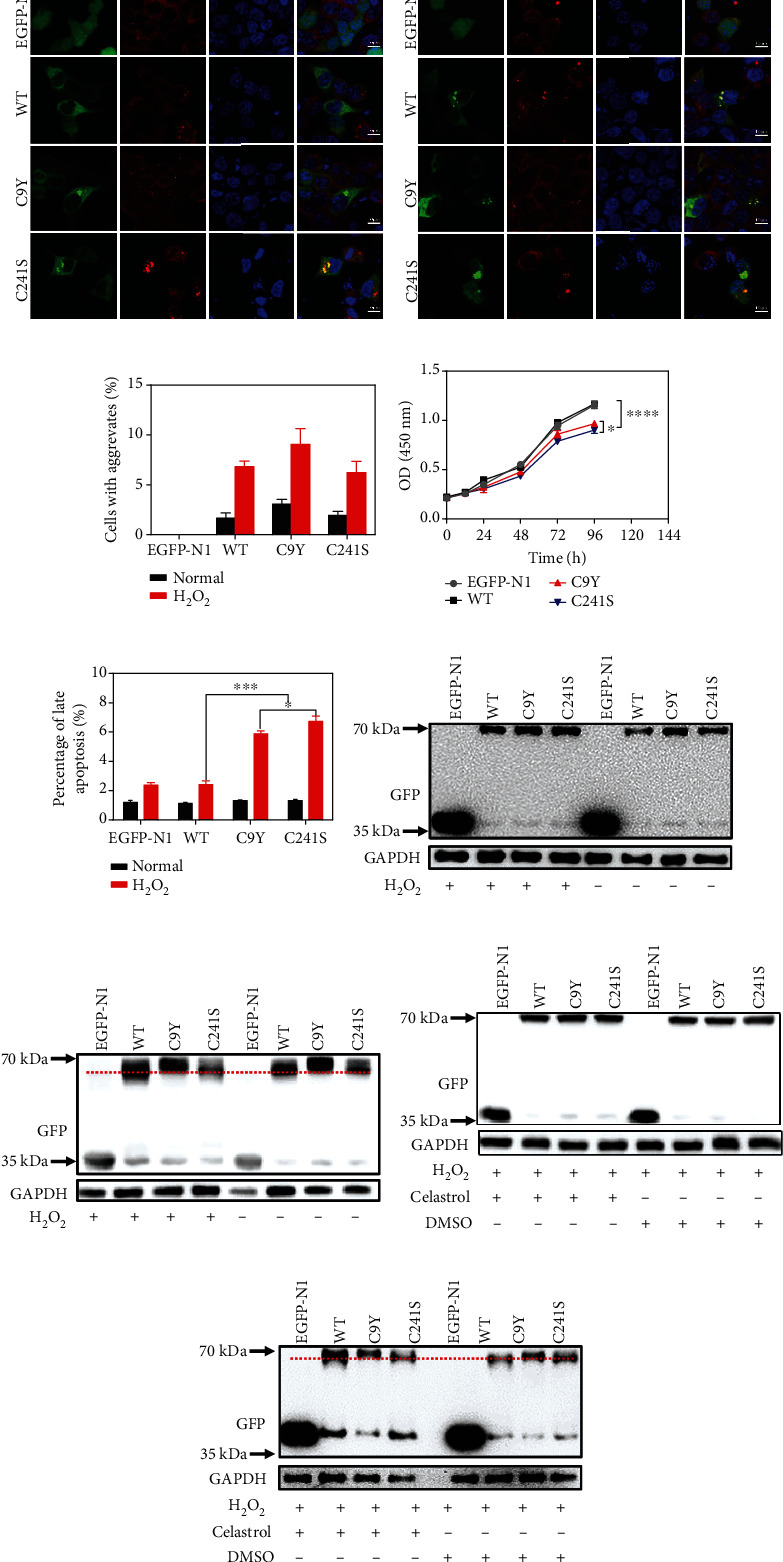
The oxidization and mutation promote protein aggregation and cell apoptosis in cell. (a, b) Representative confocal images of HEK293T cells with exogenous expression of the WT, C9Y, and C241S fused by GFP at the C-terminus without (a) or with (b) 2 mM H_2_O_2_ treatment. Exogenously expressed PMM2 proteins were visualized by GFP (green), the aggresomes were marked by P62 (red), and nucleus was stained by DAPI (blue) represented nucleus; scale bars, 10 *μ*m. (c) Quantitative analysis of protein aggregation in HEK293T cells. (d) Cell growth assay was performed in 96-well format using the CCK8 cell proliferation kit. Each sample was assayed in triplicates for 4 days consecutively. (e) Quantitative analysis of the percentages of late apoptosis. (f, g) Reduced (f) and nonreducing (g) SDS-PAGE and Western blot analysis of protein solutions without or with 2 mM H_2_O_2_ for 2 h before cells harvested. ^∗^*p* < 0.05. (h, i) Reduced (h) and nonreducing (i) SDS-PAGE and Western blot analysis of protein solutions with 2 mM H_2_O_2_ and treated with 0.5 *μ*M celastrol (^∗∗∗^*p* < 0.001, ^∗∗∗∗^*p* < 0.0001).

To provide deeper insight into the molecular pathogenesis of the Cys variants, we studied the effect at the cellular level. It was feasible to hypothesize that these proteins were susceptible to be rapidly aggregated in cells. To test our hypothesis, we overexpressed the WT and Cys variants in HEK293T cell lines. Immunofluorescence results indicated that most of the overexpressed WT and Cys variants were distributed in the cytoplasm. However, a few cells were found to form protein aggregates, which were colocalized with P62 (Figure 5(a)). As expected, the aggregates were significantly increased after 2 mM H_2_O_2_ treatment, thereby suggesting that the Cys variants were more sensitive to oxidative conditions (Figures 5(b) and 5(c)). This result was consistent with that for the purified protein in a tube. To explore the effect of Cys variants on proliferation and apoptosis, we performed CCK-8 and flow cytometry experiments. The cell growth result indicated that the proliferation abilities of the Cys variants were significantly weaker after Cys substitution and that C241S was heavier than C9Y (Figure 5(d)). Furthermore, the proportion of late apoptosis was significantly increased in the C9Y and C241S groups (Figure 5(e)). According to the WB results of reduced SDS-PAGE, after treatment for 2 h in the presence or absence of H_2_O_2_, the Cys variants did not exhibit changes in protein molecular weight (Figure 5(f)). According to the WB results of nonreduced SDS-PAGE, notably, the protein size of C9Y was larger than that of the WT, while the protein size of C241S was smaller than that of the WT (Figure 5(g)). Taken together, these findings demonstrated that the Cys substitution elevated the PMM2 susceptibility to H_2_O_2_ treatment at both protein and cellular levels.

Gámez et al. have proposed that PMM2-CDG is a conformational disease and, based on this, suggested that pharmacological chaperones may be an effective treatment [16]. Their latest work indicated that celastrol treatment led to significant increases in variant PMM2 protein concentration and activity [7]. However, in our cases, at the cellular level, during H_2_O_2_ treatment, the protein levels of Cys variants did not change after celastrol treatment, according to both reduced SDS-PAGE and nonreduced SDS-PAGE (Figures 5(h) and 5(i)). Celastrol may act as a proteostasis regulators by triggering the HSR (i.e., it increased the expression of Hsp27, Hsp40, Hsp70, and Hsp90) and by inhibiting the proteasome system, whereas knockdown the HSR led to different responses of the variants, which suggest different cellular strategies exist for dealing with misfolded proteins. In our cases, C9Y and C241S did not change the protein level compared to WT both at normal and H_2_O_2_ treatment, and celastrol did not elevate the protein level of these variants. Furthermore, we found celastrol may bind at the interface between the domains of PMM2, which is similar to the binding site of Glc-1,6-P2, the essential activator of PMM2 (supplement Figure 2). Celastrol could not change the states of Cys variants, which was taken for granted.

### 3.5. The Cysteine Variants Led to More Flexible Structure Identified by MD

**Figure 6:**
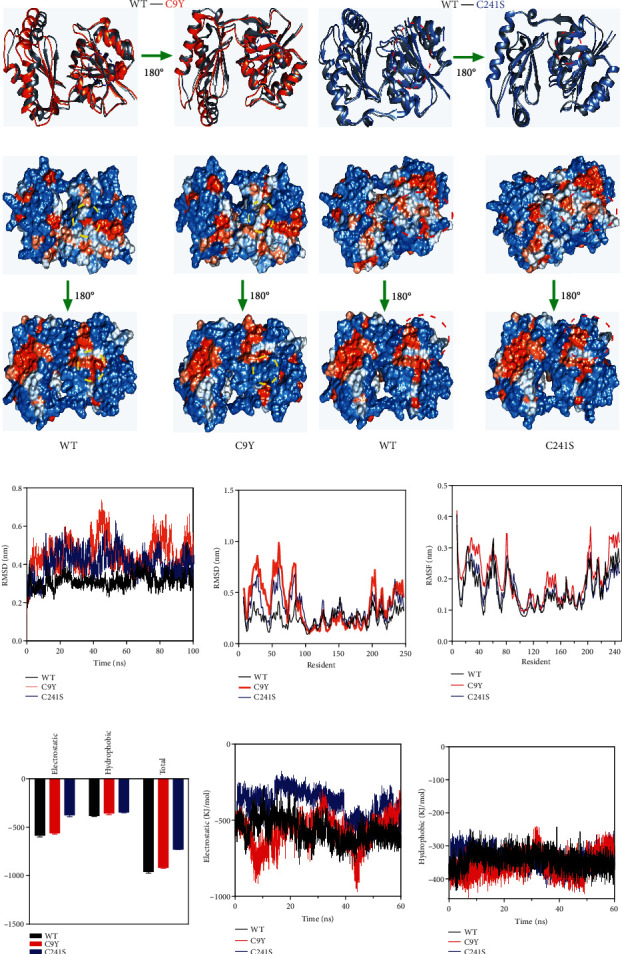
Overall dynamics of PMM2 calculated from simulations. (a) A comparison of the well-done simulated dimeric structures of the WT, C9Y, and C241S. The Cys variants altered the structure were indicated by dotted circles; blue: C9Y; red: C241S. (b) Surface electrostatic potentials of the dimeric WT and Cys variants. The Cys variants altered the distribution of charged residues, and the representative areas were indicated by dotted circles; yellow: C9Y; red: C241S. (c) C*α* root mean square deviation (RMSD) of the dimeric WT and Cys variants from the initial structure of time for all simulations. (d) RMSD of residues for all simulations. (e) C*α* root mean square fluctuation (RMSF) of residues for all simulations. (f) Average subunit binding energies calculated from the simulated structures. (g, h) The time-course changes in subunit binding energies of electrostatic (g) and hydrophobic (h).

The Cys9 and Cys241 were located in the N-terminal first *β*-sheet and last *α*-helix, respectively, in the core domain. To monitor the aggregation process caused by Cys variants, molecular dynamic (MD) simulations of the dimer was performed to establish a structural basis for the harmful effects of Cys substitution. Alignment of the dimeric structures accomplished by simulations of PMM2 and Cys variants indicated that the mutation apparently altered the overall folding of PMM2 (Figure 6(a)). A close inspection of the surface electrostatic potentials indicated that the Cys variants modified the distribution of charged/polar residues around the subunit interface, especially within the field of dashed circles (Figure 6(b)). The root mean square deviations (RMSDs) of C*α* atoms were calculated (residues 5–244 aa) from the starting structure. As shown in Figure 6(c), the RMSD of the PMM2 WT stayed fairly low, whereas the Cys variants C9Y/C241S displayed changeable values throughout the simulation. The changes were most obvious in the simulation around the C9Y and C241S mutation sites, particularly for the core domain (1-83 and 189-246 aa). The results of the time course of C*α* RMSD were similar and that of the WT remained practically steady in the last 100 ns. However, the C*α*RMSD values of C9Y and C241S varied substantially during the simulation (Figure 6(d)). Similar to the changes in the global dynamics, the C*α* root mean square fluctuations (RMSFs) from the initial structures were measured throughout the trajectories. As Figure 6(e) showed, the large changes in RMSF were focused on the local mutation sites. Furthermore, the Cys variants greatly reduced the subunit binding energy arisen from electrostatic interactions (Figures 6(f)–6(h)). Our simulation results were consistent with experimentally observed phenomena and further explained the instability of the Cys variants.

## 4. Discussion

Glycosylation modifications are ubiquitous in biology and play a pivotal role that includes recognition in the immune system and mediation of diverse responses such as cellular trafficking, and surface receptor signaling dynamics to modulate signal transduction, apoptosis, and tumor metastasis [39]. PMM2-CDG is a rare autosomal recessive disease. Normally, it is an outcome of high-risk pregnancy; the risk of having a child with PMM2-CDG is close to 1/3 instead of the expected 1/4 that was usually estimated by the previous studies [1, 19]. PMM2 is a key enzyme in the initial steps of N-glycosylation, which is essential for the translation of mannose-6-phosphate into mannose-1-phosphate [3]. Its mutation in humans leads to various kinds of diseases, including PMM2-CDG, glaucoma, hyperinsulinemic hypoglycemia, polycystic kidney disease, and premature ovarian insufficiency [40–43]. The clinical presentation and onset of PMM2-CDG vary among affected individuals according to mutation sites and types [44]. However, currently, no suitable treatment is available, only symptomatic therapy. Previous studies that focused on hotspot pathogenic variants R141H and F119L in the native state were limited to the protein level [8, 14]. Rare attention has been given to the unique Cys residue functions under stress conditions in PMM2. However, the Cys residues are active components of catalytic, oxidation-reduction, and signal transduction pathways and have distinct physicochemical properties [24]. Cys could help constrain any structural component of a peptide by creating disulfide bonds that increase the rigidity [45]. Variants in Cys have been found in various diseases, and Cys residues have potential for use as therapeutic targets [46–49]. Therefore, the goal of this article was to elucidate the molecular mechanism underlying the loss-of-function of the Cys pathogenic variants, by evaluating the pathogenicity of the Cys changes and their effects on the stability of the PMM2 protein.

Our experimental results showed that the two examined Cys pathogenic variants changed the secondary and tertiary structures, led to a looser global structure, and reduced the solubility of PMM2, as indicated by spectroscopy experiments using purified recombinant proteins (Figures 1(b)–1(g)). Concomitantly, the MD results suggested that the two Cys variants mainly disrupted the core domain (1-83 and 189-246 aa) (Figures 6(c)–6(e)). Regardless of whether the mutation was of the N-terminus (C9Y) or the C-terminus (C241S), the C*α* RMSF values fluctuated similarly, thereby suggesting that there might be a link between C9 and C241. Alignment of well-balanced simulated structures indicated that the Cys variants exhibited a higher ratio of *β*-sheet and hydrophobic surface and were prone to form aggregates, which was consistent with the Far-UV data (Figures 1(d), 1(e), 6(a), and 6(b)). As for the subunit binding energy, C241Y significantly reduced the subunit binding energy, especially in the electronic part (Figures 6(f)–6(h)). Combined with the structural analysis of the Cys variants by spectroscopy experiments and MD, these experiments provided detailed insights into the mechanism by which the Cys pathogenic variants changed the molecular structure of PMM2.

Cys residues play a vital role in sensing and protecting cells against oxidation, which is one of the major and most studied mechanisms. Oxidative stress has been established as a primary source of various forms of cellular damage, which all might result in protein misfolding and aggregation [50]. Sulfur-containing Cys residues not only manifest potent nucleophilicity but also undergo a facile oxidation reaction to generate disulfide bonds. From the structure obtained by X-ray diffraction, we measured the distance between Cys9 and Cys241, which is suitable for the formation of disulfide bonds. The result of disulfide linkages characterized by MS showed that C9 and C241 had a high potential to form intradisulfide bond (Table 1). Intrachain disulfide bonds are buried between the two layers of antiparallel *β*-sheet structure [51], and disruption the disulfide bond may explain that the C9Y and C241S destabilized the secondary and tertiary structures of PMM2. The free thiol measurement indicated that C9Y variant contained more number of free thiol than the WT, and the introduced free thiol may have come from the disruption of the disulfide bond (Figures 1(a) and 1(h)). Moreover, as shown in supplement Figure 3, after 100 ns simulation, the C9Y had a visible looser structure than WT and C241S. Meanwhile, for WT, there were two hydrogen bonds of Cys9 and two hydrogen bonds of Cys241; for C9Y and C241S, there were a total of three hydrogen bonds. The free thiol contents and hydrogen bond of the variants may explain why C9Y had less enzymatic activity than C241S and why C241S presented a milder phenotype [14, 15].

Consistent with previous results, oxidative stresses modified the structure and decreased the stability of the Cys variant proteins [47, 52]. The perturbation of the disulfide-bonding network favored hydrophobic side chain exposure, which was consistent with a previous study [46]. H_2_O_2_, considered an important redox signaling molecule, could cause oxidation of thiol groups of Cysteines in target proteins [53]. Substitution by Cys has been shown to increase sensitivity to oxidative stress in various diseases [54–56]. In our case, conversely, the Cys residues were replaced with other amino acids. Surprisingly, however, at both the purified protein level and the cellular level, we found the Cys mutation increased the susceptibility to oxidase stress (Figures 4 and 5). After H_2_O_2_ treatment, the C9Y formed more of the larger oligomers observed in the nonreducing PAGE gels. To the best of our knowledge, this is the first study to demonstrate that PMM2 would form aggregates in cells. The Cys variants could inhibit cell growth and promote cell apoptosis under oxidizing stress. It may provide a new horizon that PMM2 mutation may not only changed the enzymatic activity of PMM2 but also affected the cell viability. Of course, it is possible that decreased cell viability is the indirect result of decreased enzymatic activity.

Previous thermal stability results indicated that PMM2 variants were less stable than the WT [15]. In our work, we obtained a similar conclusion: The *A*_400_ turbidity curve showed a significant increase over time and plateaued at 4 h (Figure 2(a)). Trp and ANS fluorescence curves indicated that a large hydrophobicity surface was exposed after 37°C treatment (Figures 2(b) and 2(d)). Far-CD spectra result suggested that most of the variants lost their fold structure at physiological temperature, namely, 37°C (Figure 2(c)). Most of the Cys variants were unstable and were present in the precipitated fraction after treatment at 37°C for 4 h (Figures 2(e) and 2(f)). Further thermal aggregation results and Uncle data showed the Cys variants disrupted the compact domain organization and that the variants were prone to forming more massive aggregates (Figure 3). Meanwhile, we tested the protein stability in vivo by cycloheximide and got similar results that the C9Y and C241S mutants showed faster degradation rate than that of EGFP-N1 and WT in HEK293T cells (supplement Figure 4). Combined with the spectroscopic results shown in Figures 1 and 6, it is possible that the Cys mutation destabilized the core domain of PMM2.

Approximately 20% of human proteins are predicted to contain a disulfide bond [57]. Combined with the free thiol assay results and susceptibility to oxidative stresses, the changes that were observed in the stability and folding of the PMM2 protein in response to rupture of the disulfide bond were similar to those of some prion proteins [46]. We hypothesized that the disulfide bond in C9 and C241 stabilized the core structure much like the pincers of a crab. When one of the Cys residues was substituted, the core domain became loose, as indicated by the experimental data. The number of free thiols for C241S did not change, and C241S presented a milder clinical phenotype than C9Y [21]. This may have been due to the occurrence of unwanted intermolecular disulfide bonding as C241 was located at the C-terminus. From the MS data, for C241S variant, we guessed C9 may form disulfide bond with C136. That could explain why C241S had a free thiol content that was similar to that of the WT (Table 1). Furthermore, the potential disulfide bonding could explain why C9Y and C241S were not in accord with the expectation that the Cys residues would become more sensitive to oxidative stresses. In this case, the disulfide bonds were covalent bonds between sulfur atoms of cysteine residues, which could stabilize the structure of PMM2.

Patients with PMM2-CDG have a life-threatening insufficiency; thus, more effective drugs warrant to be developed. However, celastrol, recently proposed as a potential rescuer of PMM2 activity [7], did not change the Cys variants protein level in either nonreducing PAGE gels or reducing PAGE gels (Figure 5). And our results of molecular docking simulation revealed that celastrol did not interact at the mutation sites and no protective effects on the Cys variants. In this project, we have documented for the first time that PMM2 variants form aggresomes, inhibit cell growth, and promote cell apoptosis, especially under environmental stress (thermal and oxidative). Furthermore, we proposed Cys and potential disulfide bond may have a significant effect on the conformation and thermal stability of the PMM2. These results provide proof-of-concept regarding the clinical treatment of PMM2-CDG. Beyond pharmacological chaperones, combinations with antioxidation reagents merit investigation as treatments. Our study contributed to fill in the knowledge gap in terms of PMM2 mechanisms, and accordingly, the early detection of patients at risk and development of prevention and treatment strategies could be conducted by future studies.

## Figures and Tables

**Table 1:** Mass spectrometry (MS) detection the formation of disulfide bond.

Peptide	Proteins	Protein_Type	Score (<1.00*E* − 03)
AAPGPALCLFDVDGTLTAPR(8)-ICELLFS(2)	PMM2(9)-PMM2(241)/	Intraprotein	5.01*E* − 04
AAPGPALCLFDVDGTLTAPR(8)-SCSQEER(2)	PMM2(9)-PMM2(136)/	Intraprotein	5.31*E* − 05
QNIQSHLGEALIQDLINYCLSYIAK(19)-ICELLFS(2)	PMM2(103)-PMM2(241)/	Intraprotein	4.55*E* − 07
QNIQSHLGEALIQDLINYCLSYIAK(19)-SCSQEERIEFYELDKK(2)	PMM2(103)-PMM2(136)/	Intraprotein	5.31*E* − 05
SCSQEER(2)-ICELLFS(2)	PMM2(136)-PMM2(241)/	Intraprotein	6.19*E* − 06
SCSQEERIEFYELDKK(2)-SCSQEERIEFYELDKK(2)	PMM2(136)-PMM2(136)/	Interprotein	7.69*E* − 07

## Data Availability

The original data used to support the findings of this study are available from the corresponding author upon request.

## Supplementary Materials

Supplementary Materials Supplement Figure 1: analysis the enzymatic activity of purified WT-PMM2. Supplement Figure 2: the molecular docking simulation of celastrol by AutoDock Vina software. Supplement Figure 3: analysis the structural difference by MD. (A) The last frames of the MD equilibrations were used as structures for further analysis. (B) Analysis the hydrogen bonding of Cys9 (shown with green color) and Cys241 (shown with red color). Supplement Figure 4: the CHX-mediated protein stability assay of EGFP-N1, WT, C9Y, and C241S mutants. (A) Cells were harvested at the indicated times (0, 2, 4, 6, 8, and 12 h) after treatment of 100 *μ*g/mL CHX and analyzed by Western blotting of GFP. GAPDH was used as a loading control. (B) The relative amounts of GFP for the Western blots shown in (A) were determined by densitometry and corrected for loading against GAPDH. The ratio of relative densitometry value at each time to 0 h is presented on the line chart.
