# Supplementary material for: Cysteine Pathogenic Variants of PMM2 Are Sensitive to Environmental Stress with Loss of Structural Stability
Source: Oxid Med Cell Longev. 2023 Jan 25;2023:5964723. doi: 10.1155/2023/5964723 (PMC9891822; doi:10.1155/2023/5964723)
Supplement: Supplementary Materials — Supplement Figure 1: analysis the enzymatic activity of purified WT-PMM2. Supplement Figure 2: the molecular docking simulation of celastrol by AutoDock Vina software. Supplement Figure 3: analysis the structural difference by MD. (A) The last frames of the MD equilibrations were used as structures for further analysis. (B) Analysis the hydrogen bonding of Cys9 (shown with green color) and Cys241 (shown with red color). Supplement Figure 4: the CHX-mediated protein stability assay of EGFP-N1, WT, C9Y, and C241S mutants. (A) Cells were harvested at the indicated times (0, 2, 4, 6, 8, and 12 h) after treatment of 100 μg/mL CHX and analyzed by Western blotting of GFP. GAPDH was used as a loading control. (B) The relative amounts of GFP for the Western blots shown in (A) were determined by densitometry and corrected for loading against GAPDH. The ratio of relative densitometry value at each time to 0 h is presented on the line chart. [file 5964723.f1.docx]

**Materials and methods**

## Enzyme assay

The PMM activity was assayed spectrophotometrically at 340 nm by the reduction of NADP+ to NADPH in a reaction mixture incubated at 30℃ for indicated times (0, 1, 2, 3, 4, 5 min). The reaction mixtures contained 50 mM Hepes (pH 7.1), 5 mM MgCl_2_, 0.25 mM NADP+，10 μg/ml yeast glucose 6-phosphate dehydrogenase，0.1 mM mannose 1-phosphate, 10 μM glucose 1,6-bisphosphate, 10 μg/ml phosphoglucose isomerase and 3.5 μg/ml phosphomannose isomerase.

## Molecular docking simulation

The molecular docking simulation of celastrol which reported as drug of PMM2 was performed through AutoDock Vina software. Prior to docking, the 3D structure of PMM2 was prepared using the Structure Preparation module in AutoDock by detecting candidate protein-ligand and protein-protein binding sites using a fast geometric algorithm based on Edelsbrunner's Alpha Shapes. The default parameters in AutoDock were used in docking program. After docking preparation, we got "dummy atoms" for docking calculations or starting points for de novo ligand design efforts. Then we import the 3D structure of celastrol molecular (download from ZINC15). Finally, we obtained 100 docking poses.

## Cycloheximide-decay assay

After transient transfection for 24 h, different group of cells were seeded to 12-well plates. When the cells were 80 % confluent, the cells were treated with fresh medium containing 100 μg/ml cycloheximide (CHX). Cells were collected at the indicated times (0, 2, 4, 6, 8, 12h) after treatment CHX and used to do western blot after lysed.

**supplement Figure 1.**

**supplement Figure 1.** Analysis the enzymatic activity of purified WT-PMM2.

**supplement Figure 2.**


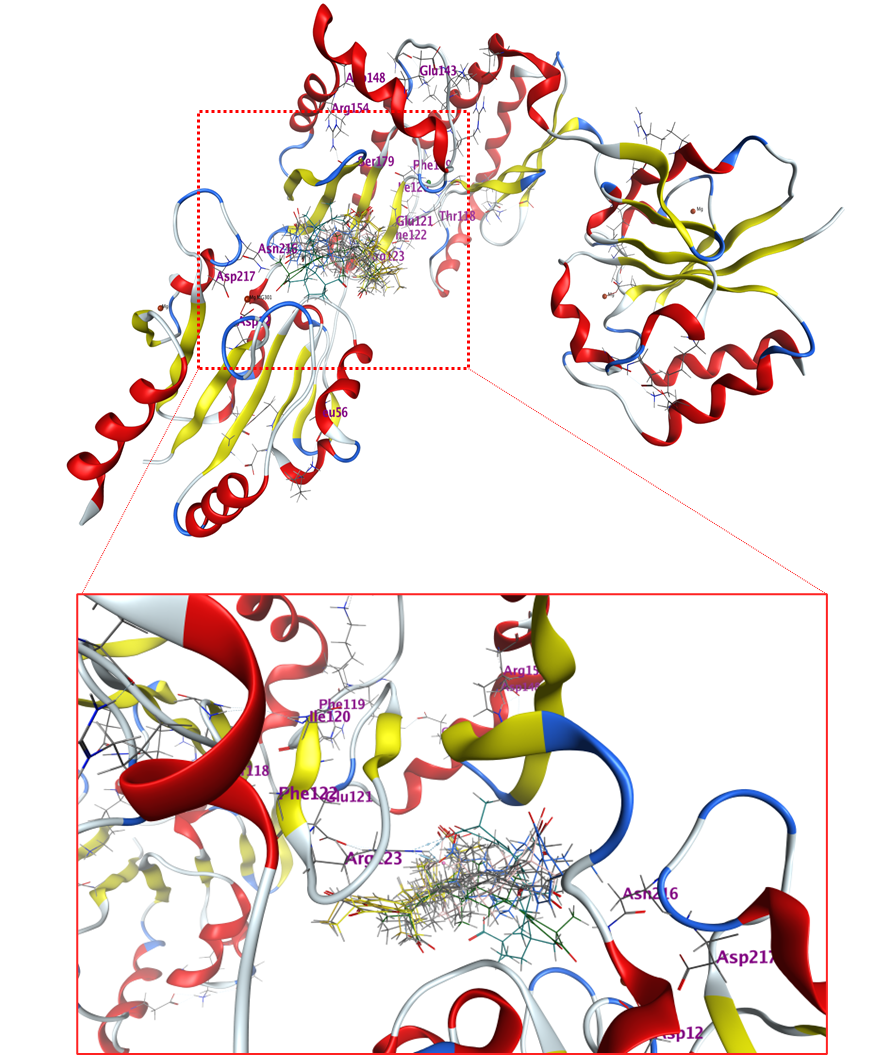


**supplement Figure 2.** The molecular docking simulation of celastrol by AutoDock Vina software.

**supplement Figure 3.**


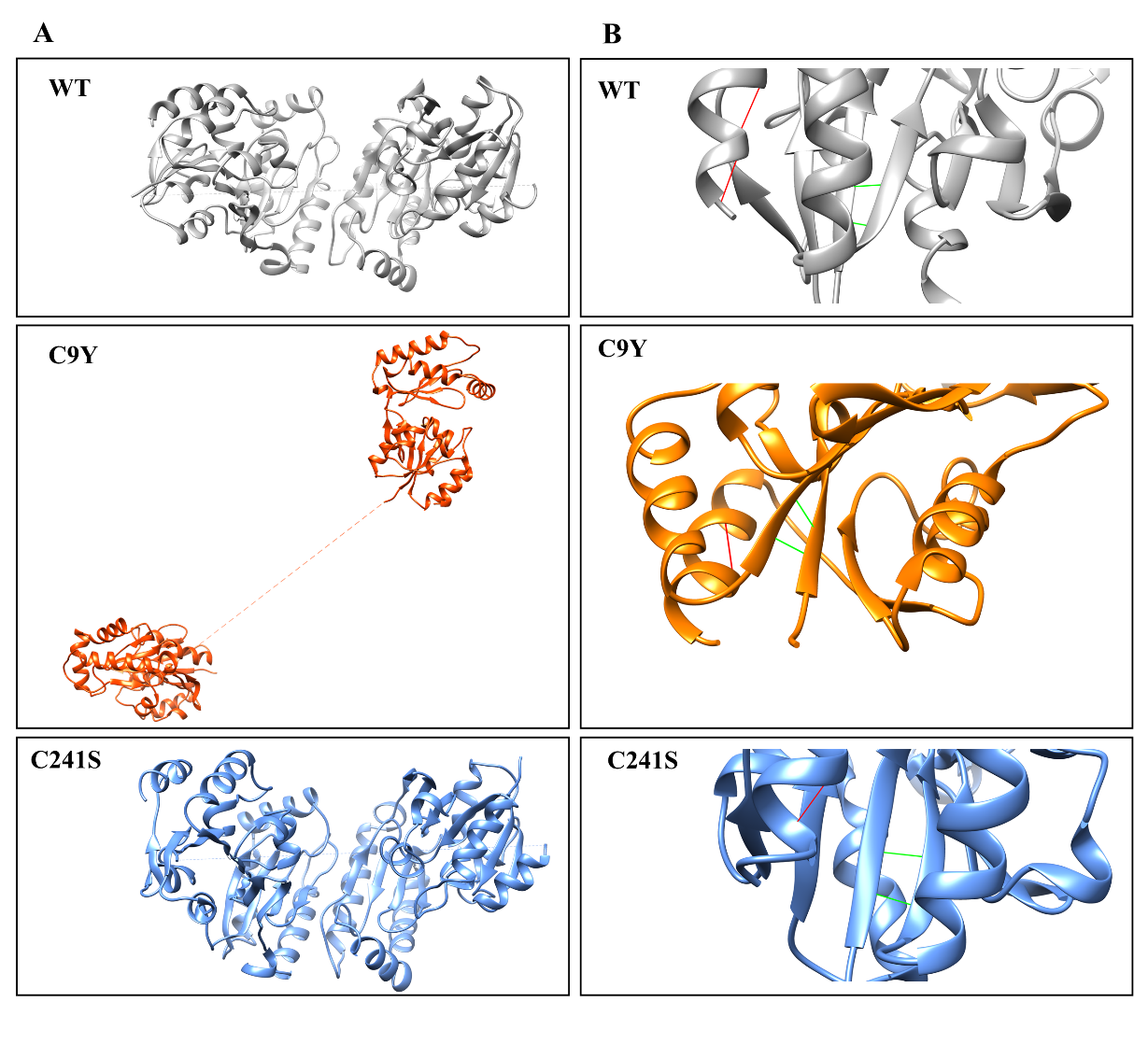
 **supplement Figure 3.** Analysis the structural difference by MD. **(A)** The last frames of the MD equilibrations were used as structures for further analysis. **(B)** Analysis the hydrogen bonding of Cys9 (shown with green color) and Cys241 (shown with red color).

**supplement Figure 4.**


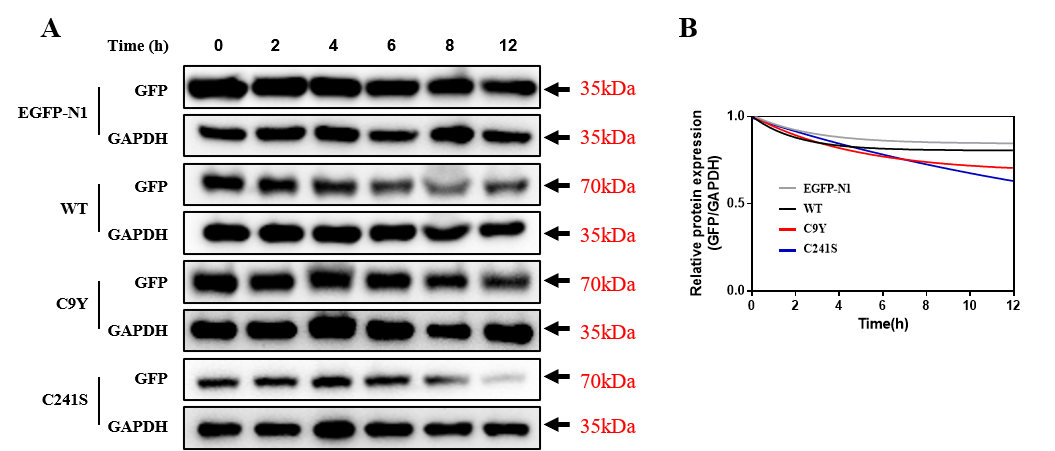


**supplement Figure 4.** The CHX mediated protein stability assay of EGFP-N1, WT, C9Y and C241S mutants. **(A)** Cells were harvested at the indicated times (0, 2, 4, 6, 8, 12 h) after treatment of 100 μg/ml CHX and analyzed by Western blotting of GFP. GAPDH was used as a loading control. **(B)** The relative amounts of GFP for the Western blots shown in A were determined by densitometry and corrected for loading against GAPDH. The ratio of relative densitometry value at each time to 0 h is presented on the line chart.
